# Blood urea nitrogen and bladder cancer: A Mendelian randomization study

**DOI:** 10.1097/MD.0000000000046046

**Published:** 2026-05-12

**Authors:** Jinming Bai, Lingling Wang

**Affiliations:** a Department of Neurology, Stroke Center, The Affiliated Hospital of Beihua University, Jilin, Jilin Province, China.

**Keywords:** bladder cancer, blood urea nitrogen, gene, Mendelian randomization analysis

## Abstract

The causal relationship between blood urea nitrogen (BUN) level and bladder cancer (BC) was analyzed by 2-sample Mendel randomization method, in order to provide help for the screening of high-risk groups of BC. Based on the summary data of whole genome association analysis, and Mendelian randomization (MR) is performed using inverse variance weighted (IVW), weighted median (WME), and MR Egger regression methods. IVW analysis is the main result, supplemented by WME and MR Egger regression results. Cochran Q statistics, MR Egger intercept term, MR-PRESSO test, leave 1 out method, and funnel plot are used for sensitivity analysis to ensure the robustness of the analysis results. A total of 178 single nucleotide polymorphisms strongly associated with BUN level were included. IVW analysis showed that there was a causal relationship between blood ureanitrogen and BC (OR = 1.0016, 95% *CI*: 1.0004–1.0027, *P* <.05). WME analysis results supported the above conclusions, but MR Egger regression analysis did not show a causal relationship between the 2. Cochran *Q* statistic *P* >.05, MR Egger intercept method (*P* = .63), MR-PRESSO test (*P* = .36), “leave one out” method, and funnel plot analysis all indicate that there is no heterogeneity or horizontal pleiotropy in the MR analysis of the 2 samples, and the analysis results are stable and reliable. There is a causal relationship between high BUN levels and BC, which provides reference for prevention and treatment of BC.

## 1. Introduction

Bladder cancer (BC) is one of the most common tumors in the urinary system, with a high incidence rate and high mortality. It is reported that in 2022, the incidence rate of BC will be the sixth in the incidence rate of various cancers, and the mortality will be the ninth. With the change of people’s diet structure and lifestyle, its incidence rate will continue to increase year by year. The histopathological types of BC include urothelial carcinoma, squamous cell carcinoma, adenocarcinoma, etc urothelial carcinoma accounts for more than 90%, which is the most common type of BC cancer.^[1]^ The disease only shows painless hematuria in the early stage. As the condition worsens, symptoms such as frequent urination, urgency, and pain gradually appear. If not treated in time, bladder function may be permanently lost, or even distant metastasis may occur, endangering life.^[2]^ Therefore, more attention should be paid to BC at home and abroad, and early prevention, early detection and early treatment should be achieved for BC to reduce the pain of patients. Blood urea nitrogen (BUN) is the end product of protein metabolism. Amino acids in the body are deaminated and broken down into alpha keto groups and NH3, which are then converted into urea in the liver and CO2. Urea is then excreted in the urine through glomerular filtration and tubular reabsorption. Therefore, the level of BUN is related to protein intake, tissue protein catabolism, liver function, and glomerular filtration function. In clinical practice, this indicator is often used to evaluate renal function.

Mendelian randomization (MR) is a research method that uses genetic variations strongly associated with exposure factors as instrumental variables (IV), namely single nucleotide polymorphisms (SNPs), to analyze whether there is a causal relationship between exposure and outcomes.^[3]^ Compared with observational studies and randomized controlled trials, its advantage is based on the random allocation of alleles during meiosis, which can avoid the drawbacks of traditional confounding factors and reverse causality. Therefore, it has become a new method for exploring the causal relationship between risk factors and diseases. In addition, MR research uses R software to obtain publicly available data from databases, so there is no need for additional ethical review, which saves time and manpower compared to previous research methods. In recent years, domestic and international scholars have utilized MR studies to identify correlations between blood and urine biochemical indicators and urinary tract tumors. Current research hotspots include the correlation between serum calcium levels and sex hormone-binding globulin with renal cancer.^[4,5]^ However, studies investigating the causal relationship between BUN and BC remain limited. Therefore, this study uses MR research to analyze the causal relationship between BUN level and BC from the perspective of gene, in order to provide reference for early prevention, intervention and treatment of BC.

## 2. Methods

### 2.1. Research design and data sources

**Figure 1.**
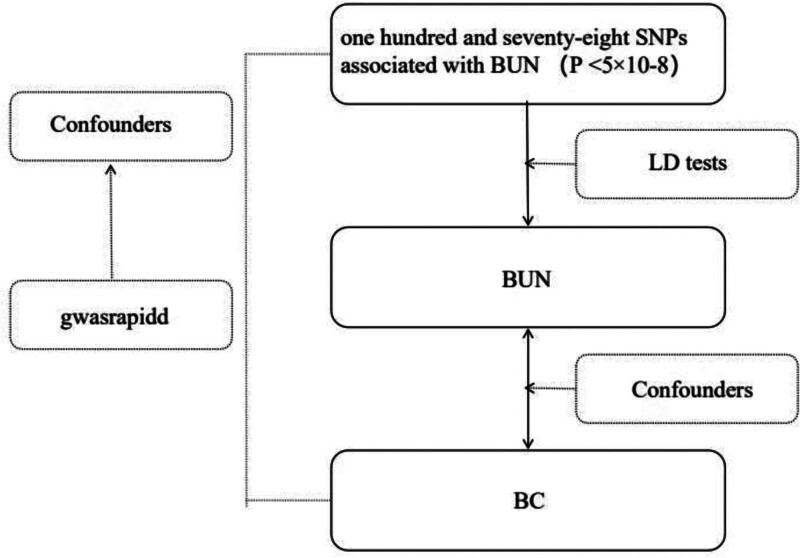
BUN and BC Mendel randomized analysis flow chart. BC = bladder cancer, BUN = blood urea nitrogen.

This study employed a 2-sample MR analysis to explore the causal relationship between BUN and BC, followed by reverse analysis in strict compliance with the STROBE-MR guidelines (see Supplementary STROBE-MR Checklist, Supplemental Digital Content, https://links.lww.com/MD/Q783). The exposure factors, IV, and outcome variables were: BUN levels, SNPs strongly associated with BUN levels (*P* < 5 × 10^-8), and BC. To ensure reliability of the analysis, all genetic variants must satisfy 3 MR study assumptions: IV must have a strong correlation with the exposure factor (BUN level); The selected IV must not correlate with confounding factors; The IV must not influence the outcome variable (BC) through other pathways but can only affect it via the exposure factor, thereby avoiding level multiplicity. The workflow diagram is shown in Figure 1.

The summary data of BUN comes from the IEU open GWAS database(https://gwas.mrcieu.ac.uk/datasets/ebi-a-GCST90018948/)The total sample size is 344,052 cases, with a total of 19,049,084 SNPs. Summary data of BC is from IEU open GWAS database(https://gwas.mrcieu.ac.uk/datasets/ieu-b-4874/)The total sample size is 373,295 cases, including 1279 cases in the experimental group and 372,016 cases in the control group, with a total of 9,904,926 SNPs. The above 2 sets of data are both of European descent, avoiding bias in results caused by racial factors. The data in this study were obtained from public databases using R software and do not require additional ethical approval. GWAS database research data information.

### 2.2. Selection and handling of instrumental variables

To meet the 3 hypotheses of MR, this study will strictly screen IV according to the criteria Set the screening criteria as follows: *P* < 5 × 10-8, r2 < 0.001, M > 10000kb, where P is the standard threshold. When *P* < 5 × 10-8, it indicates a significant correlation between SNPs and exposure factors (BUN levels); R2 is data between 0 and 1, and the smaller its value, the more completely random the allocation of 2 SNPs is; M represents the length of the linkage disequilibrium. When r2 < 0.001 and M > 10000kb, the screened SNPs have independent heritability To avoid bias in MR research results caused by the use of weak tools, an F-value > 10 is defined as having no weak tool bias to ensure a strong correlation between the selected SNPs and BUN levels. The F-value calculation formula is: F=β 2/SE2, where β represents the effect value of BUN related SNPs and SE represents the standard error of β. After calculation, the strongly correlated SNPs with F > 10 are retained In order to ensure that the effective alleles of BUN and BC are consistent, palindrome sequences that cannot determine the direction are removed. After the above steps of screening and processing, the SNPs obtained will be included in this MR study (Table 1).

**Table 1:** The detailed information of identified SNPs in exposure and outcomes.

SNP					Exposure (BUN)				Outcome (BC)				*F*
RS ID	Chromosome	Position	EA	OA	Beta	Se	*P*-value	EAF	Beta	SE	*P*-value	EAF	
rs10224210	7	151,413,194	C	T	0.0492	0.0025	4.88203 × 10^−83^	0.28248	−6.08 × 10^−05^	0.000150284	0.689999855838142	NA	387.3024
rs10440057	3	186,434,108	A	G	0.0118	0.002	9.2619 × 10^−09^	0.309696	−6.76 × 10^−05^	0.000145243	0.640000038338762	NA	34.81
rs1047891	2	211,540,507	A	C	−0.0318	0.0022	1.97606 × 10^−47^	0.26612	−0.000137687	0.000145286	0.340000064945877	NA	208.933884297521
rs10766469	11	18,323,316	C	G	0.0106	0.0019	3.88803 × 10^−08^	0.574828	7.9743 × 10^−05^	0.000136816	0.559999965176919	NA	31.1246537396122
rs10767859	11	30,571,347	T	C	0.0124	0.0019	1.41801 × 10^−10^	0.465138	0.000187506	0.000137651	0.170000030775649	NA	42.5927977839335
rs10796869	11	69,216,024	T	C	0.0134	0.0019	5.99239 × 10^−12^	0.486974	−4.04 × 10^−05^	0.000138211	0.770000487266739	NA	49.7396121883657
rs10863888	1	211,502,769	G	A	0.0127	0.0019	5.74513 × 10^−11^	0.596803	0.000159607	0.000138552	0.249999995007974	NA	44.6786703601108
rs10892247	11	118,490,076	A	G	0.014	0.0023	2.85503 × 10^−09^	0.20798	−7.37 × 10^−05^	0.000162394	0.649999466219879	NA	37.0510396975425
rs10929831	2	12,924,836	G	A	−0.0114	0.002	9.39399 × 10^−09^	0.569659	−7.35 × 10^−06^	0.000135217	0.959999926966642	NA	32.49
rs10935972	3	153,774,029	A	G	−0.0187	0.0032	6.56206 × 10^−09^	0.1511	−0.000209337	0.000190903	0.27000014662192	NA	34.14,941,406
rs10937339	3	187,775,100	G	A	−0.0346	0.0032	2.32702 × 10^−27^	0.84418	3.54631 × 10^−05^	0.000188953	0.849999949672056	NA	116.9101563
rs11000927	10	75,979,691	G	T	−0.0173	0.0021	6.18301 × 10^−17^	0.647043	1.92375 × 10^−05^	0.000153622	0.899999980438636	NA	67.8662131519275
rs11032736	11	34,538,237	C	T	−0.0142	0.0022	9.9954 × 10^−11^	0.35082	−3.01 × 10^−05^	0.000135529	0.820000089905251	NA	41.6611570247934
rs1108921	13	73,716,317	G	T	−0.0156	0.0022	2.11398 × 10^−12^	0.745205	0.000203804	0.000171173	0.230000086844209	NA	50.2809917355372
rs1113499	17	36,170,774	A	T	−0.0139	0.0021	1.75388 × 10^−11^	0.680787	−2.22 × 10^−05^	0.000140592	0.870000094916712	NA	43.8117913832199
rs11163481	1	82,937,433	T	G	0.0153	0.002	1.09094 × 10^−14^	0.640444	−0.000227617	0.000144186	0.110000079744526	NA	58.5225
rs11453704	10	60,265,164	AC	A	−0.0175	0.0019	7.73215 × 10^−20^	0.466139	0.000113936	0.00013693	0.410000135265173	NA	84.8337950138504
rs11525583	12	112,261,638	A	G	0.0192	0.0033	7.64997 × 10^−09^	0.200526	−0.000101063	0.000537776	0.849999949672056	NA	33.8512396694215
rs11574435	3	46,447,972	T	C	0.0203	0.0035	9.18502 × 10^−09^	0.12384	0.000141904	0.000208215	0.499999995007974	NA	33.64
rs11577667	1	33,914,333	G	T	−0.015	0.002	2.31579 × 10^−13^	0.326151	0.000122978	0.000145344	0.400000007987242	NA	56.25
rs11647746	16	89,806,343	A	C	0.0229	0.0032	7.11214 × 10^−13^	0.0980009	−0.000235915	0.000229701	0.299999824045942	NA	51.21191406
rs11672660	19	46,180,184	T	C	−0.039	0.0023	4.7326 × 10^−62^	0.204311	0.00015267	0.000170657	0.370000235082988	NA	287.523629489603
rs11681706	2	85,486,354	G	T	−0.014	0.0022	8.26799 × 10^−11^	0.645798	−0.000331615	0.000161111	0.0400000007987242	NA	40.495867768595
rs1169300	12	121,431,225	A	G	0.0214	0.002	4.33112 × 10^−26^	0.365795	0.000361692	0.000148566	0.0149999565138204	NA	114.49
rs11729899	4	40,436,704	G	A	0.0172	0.0023	9.07612 × 10^−14^	0.51954	−9.36 × 10^−05^	0.000135675	0.489999909706502	NA	55.9243856332703
rs117643180	17	7,185,779	A	C	0.0487	0.0066	1.80509 × 10^−13^	0.02138	−0.000478325	0.00042962	0.27000014662192	NA	54.4465105601469
rs11855188	15	77,840,714	G	A	−0.0159	0.0021	2.62603 × 10^−14^	0.675246	0.000125932	0.000155833	0.419999719160426	NA	57.3265306122449
rs11960585	5	40,662,007	A	C	−0.1103	0.0044	2.71019 × 10^−136^	0.0500817	−0.000230688	0.000262181	0.380000352953329	NA	628.413739669421
rs1200485	5	72,385,058	G	A	−0.0144	0.0019	3.93641 × 10^−14^	0.512736	0.00010852	0.000135806	0.419999719160426	NA	57.4404432132964
rs12029610	1	54,844,602	T	C	0.0232	0.0025	7.71792 × 10^−20^	0.181201	0.000112732	0.000213493	0.599999654082312	NA	86.1184
rs12117131	1	67,451,922	T	C	−0.0257	0.0024	2.60196 × 10^−26^	0.20579	−7.91 × 10^−05^	0.000153984	0.610000231740111	NA	114.668402777778
rs12151866	20	56,044,744	G	C	0.0126	0.0022	6.19298 × 10^−09^	0.26135	−9.95 × 10^−05^	0.00015593	0.519999588551012	NA	32.801652892562
rs12247543	10	126,419,743	C	G	−0.0176	0.0022	1.92708 × 10^−15^	0.245222	8.71757 × 10^−05^	0.000153252	0.570000189426062	NA	64
rs1229984	4	100,239,319	C	T	−0.0296	0.0036	3.07114 × 10^−16^	0.756667	−0.000788457	0.000407908	0.0530005040639665	NA	67.6049382716049
rs12355313	10	63,805,376	G	A	−0.0132	0.0019	9.20662 × 10^−12^	0.484833	0.000292243	0.000135951	0.0320000015974484	NA	48.2659279778393
rs12476098	2	177,077,489	C	G	0.0507	0.0063	1.11404 × 10^−15^	0.034086	0.000113326	0.000204651	0.580000008596693	NA	64.7641723356009
rs1260326	2	27,730,940	C	T	−0.0204	0.0019	3.7368 × 10^−26^	0.557206	0.000109025	0.000138107	0.429999548925608	NA	115.279778393352
rs1275609	12	76,271,183	A	G	−0.0156	0.002	1.51008 × 10^−14^	0.403117	−2.32 × 10^−05^	0.000146502	0.870000094916712	NA	60.84
rs12940987	17	59,269,257	A	G	0.0283	0.0023	4.05322 × 10^−36^	0.77008	0.000216485	0.000163816	0.190000174579693	NA	151.396975425331
rs12963357	18	43,252,053	T	C	0.1449	0.0089	1.07597 × 10^−59^	0.017224	−1.82 × 10^−05^	0.000529765	0.969999923466087	NA	265.067668223709
rs13032786	2	148,803,672	G	C	0.0139	0.002	8.91046 × 10^−12^	0.337838	−8.50 × 10^−05^	0.000148221	0.570000189426062	NA	48.3025
rs13059257	3	195,634,993	T	C	0.0443	0.0068	7.77141 × 10^−11^	0.029335	−0.00033621	0.000398194	0.400000007987242	NA	42.441392733564
rs13080378	3	66,804,200	G	A	0.0401	0.0024	3.55222 × 10^−61^	0.188033	−0.000304274	0.000171132	0.0749997818203002	NA	279.168402777778
rs13155267	5	90,193,468	C	T	−0.0147	0.002	6.95665 × 10^−13^	0.370402	−8.91 × 10^−05^	0.000150918	0.550000393231399	NA	54.0225
rs13230509	7	1,286,192	C	G	0.0499	0.0021	4.47713 × 10^−125^	0.578487	−6.11 × 10^−05^	0.000150912	0.689999855838142	NA	564.628117913832
rs13241164	7	625,830	T	C	0.0184	0.0023	2.57395 × 10^−15^	0.57003	−0.000216501	0.000137097	0.110000079744526	NA	64
rs13335361	16	69,536,919	A	G	0.0167	0.0028	3.94703 × 10^−09^	0.150131	−3.13 × 10^−05^	0.000187084	0.870000094916712	NA	35.5727040816327
rs1392969	6	101,091,434	C	T	−0.0172	0.003	1.63599 × 10^−08^	0.157383	−0.00012437	0.000419422	0.770000487266739	NA	32.8711111111111
rs146346574	15	77,169,485	A	G	−0.0535	0.0083	1.16399 × 10^−10^	0.019686	0.000124777	0.000494022	0.800000023961726	NA	41.5481201916098
rs1484873	18	43,206,985	G	A	0.0562	0.0033	6.18728 × 10^−66^	0.905136	0.000264258	0.000301538	0.380000352953329	NA	290.031221303949
rs1554447	6	154,983,792	T	C	−0.0155	0.0026	2 × 10^−09^	0.836401	−0.000400697	0.000190843	0.0359997927043357	NA	35.5399408284024
rs1595066	2	212,241,725	T	C	0.0118	0.002	2.05201 × 10^−09^	0.376112	−8.39 × 10^−05^	0.000143216	0.559999965176919	NA	34.81
rs160329	2	136,617,805	T	C	0.017	0.0028	2.117 × 10^−09^	0.78607	5.6139 × 10^−05^	0.000158574	0.719999176986299	NA	36.8622448979592
rs162004	18	24,447,936	G	C	0.0152	0.002	3.99669 × 10^−14^	0.65597	−0.000224052	0.000140945	0.110000079744526	NA	57.76
rs17050272	2	121,306,440	A	G	0.0184	0.0019	8.51334 × 10^−22^	0.431967	−2.94 × 10^−05^	0.000137493	0.830000014570433	NA	93.7839335180056
rs17216707	20	52,732,362	C	T	−0.0272	0.0027	7.2061 × 10^−24^	0.157259	−7.28 × 10^−05^	0.000175223	0.680000136680911	NA	101.486968449931
rs17398736	8	66,948,383	G	C	0.0186	0.0028	1.74502 × 10^−11^	0.145406	−0.000303261	0.000177279	0.0870001497192316	NA	44.1275510204082
rs17645325	5	158,476,709	C	T	−0.0224	0.0035	1.086 × 10^−10^	0.12528	−0.000223168	0.000205806	0.279999979792925	NA	40.96
rs17663555	5	72,432,036	G	C	0.0305	0.002	1.51286 × 10^−51^	0.32936	0.000338434	0.000144987	0.020000000199681	NA	232.5625
rs17730281	15	53,907,948	A	G	−0.0265	0.0021	3.18787 × 10^−35^	0.27638	−0.000361082	0.000161812	0.0259997995670924	NA	159.240362811791
rs186886470	2	25,946,280	C	T	0.0378	0.0066	1.14101 × 10^−08^	0.03129	−3.57 × 10^−05^	0.000390455	0.929999896026408	NA	32.801652892562
rs187355703	2	176,993,583	G	C	0.0675	0.0072	6.54485 × 10^−21^	0.018487	0.000500217	0.000432552	0.249999995007974	NA	87.890625
rs2025614	1	78,618,151	A	G	0.0112	0.002	2.62398 × 10^−08^	0.507055	−0.00011607	0.00013898	0.400000007987242	NA	31.36
rs2039424	9	71,432,174	A	G	−0.0123	0.002	6.047 × 10^−10^	0.640365	0.000177059	0.000139689	0.20000000199681	NA	37.8225
rs2156172	11	86,637,337	G	A	0.0176	0.002	2.83531 × 10^−18^	0.669674	0.000165029	0.000147221	0.259999791679655	NA	77.44
rs219779	21	37,833,751	A	G	−0.0312	0.0026	6.94864 × 10^−34^	0.186631	−0.000186449	0.000153558	0.220000161685546	NA	144
rs2332036	3	121,714,391	T	C	0.0219	0.002	3.2181 × 10^−28^	0.559332	0.000186922	0.00013624	0.170000030775649	NA	119.9025
rs2466076	8	32,432,796	T	G	−0.0119	0.002	3.095 × 10^−09^	0.617579	3.7949 × 10^−05^	0.000136261	0.780000713574729	NA	35.4025
rs2519093	9	136,141,870	T	C	0.0186	0.0023	1.58708 × 10^−15^	0.211685	−0.000189403	0.000174166	0.279999979792925	NA	65.398865784499
rs2596398	11	47,249,463	C	G	0.0182	0.0024	1.10994 × 10^−14^	0.692693	−0.000223865	0.000185934	0.230000086844209	NA	57.5069444444445
rs270164	5	139,537,032	T	G	−0.0116	0.0021	4.73598 × 10^−08^	0.353798	−7.38 × 10^−05^	0.000157162	0.640000038338762	NA	30.5124716553288
rs2749033	6	52,632,479	G	A	0.0188	0.0025	3.20332 × 10^−14^	0.781894	0.000178666	0.000146229	0.220000161685546	NA	56.5504
rs2762943	20	52,790,786	G	T	0.0256	0.0043	2.74397 × 10^−09^	0.923131	−0.000181521	0.000254368	0.479999737642877	NA	35.4440237966468
rs2800712	6	127,378,777	A	G	−0.0145	0.0024	2.19498 × 10^−09^	0.77798	−0.000144022	0.000192277	0.450000503808422	NA	36.5017361111111
rs2823139	21	16,576,783	A	G	0.0171	0.0021	2.1702 × 10^−16^	0.311549	5.33459 × 10^−05^	0.000143724	0.709999429901934	NA	66.3061224489796
rs2834310	21	35,343,934	G	A	0.0143	0.0019	8.93511 × 10^−14^	0.466695	−7.95 × 10^−05^	0.000135375	0.559999965176919	NA	56.6454293628809
rs28362590	5	176,731,452	T	G	0.014	0.0021	1.95389 × 10^−11^	0.663381	−1.35 × 10^−05^	0.000156718	0.929999896026408	NA	44.4444444444445
rs28394165	4	77,394,018	C	T	0.0268	0.002	1.27909 × 10^−40^	0.384142	−0.000229495	0.000135672	0.0909997082601283	NA	179.56
rs28712821	4	39,413,780	A	G	−0.0181	0.0019	1.7338 × 10^−20^	0.550463	8.97957 × 10^−05^	0.000139214	0.519999588551012	NA	90.7506925207756
rs2960172	17	47,379,581	A	G	0.0156	0.002	9.58959 × 10^−15^	0.328148	5.56339 × 10^−06^	0.000145758	0.969999923466087	NA	60.84
rs300143	6	166,421,127	G	A	−0.0356	0.0021	5.21315 × 10^−65^	0.343358	−0.000141907	0.000137071	0.299999824045942	NA	287.383219954648
rs3010095	1	202,171,044	G	A	0.0134	0.0022	6.52394 × 10^−10^	0.715133	−0.000294991	0.000145582	0.043000152915127	NA	37.099173553719
rs3212531	5	52,354,424	C	G	−0.0161	0.0029	3.00303 × 10^−08^	0.127118	−1.92 × 10^−05^	0.000258587	0.940000100430209	NA	30.8216409036861
rs34741056	12	122,511,754	C	T	0.0106	0.0019	2.56702 × 10^−08^	0.516993	0.000150477	0.000135585	0.27000014662192	NA	31.1246537396122
rs347593	3	11,248,957	G	C	0.0201	0.003	1.88495 × 10^−11^	0.81731	−0.00013243	0.000175968	0.450000503808422	NA	44.89
rs35601737	19	13,220,703	G	C	0.0162	0.0025	8.19219 × 10^−11^	0.30776	0.000305274	0.000146943	0.0379996853036855	NA	41.9904
rs35610898	5	176,815,766	C	G	0.0234	0.002	2.52522 × 10^−30^	0.311073	−9.05 × 10^−05^	0.000146288	0.540000298635231	NA	136.89
rs35873897	1	31,685,975	CA	C	0.016	0.0029	2.48399 × 10^−08^	0.79542	−0.000283278	0.000169632	0.0949992113622595	NA	30.4399524375743
rs35949897	12	15,346,379	T	C	−0.0164	0.0022	2.28297 × 10^−13^	0.244722	−9.85 × 10^−05^	0.000168012	0.559999965176919	NA	55.5702479338843
rs36079436	17	81,058,310	C	T	0.0116	0.002	9.52599 × 10^−09^	0.593548	0.000199031	0.000143196	0.160000006389794	NA	33.64
rs3736167	17	34,900,847	C	A	−0.0114	0.0019	3.82904 × 10^−09^	0.405969	6.54316 × 10^−06^	0.000137714	0.959999926966642	NA	36
rs3767848	1	214,173,840	A	G	−0.0165	0.0021	1.36301 × 10^−14^	0.268168	−0.000502962	0.000156204	0.00129998996537545	NA	61.734693877551
rs3791221	2	226,933	G	A	0.0253	0.0024	7.22603 × 10^−26^	0.35022	0.000183722	0.000142048	0.20000000199681	NA	111.126736111111
rs3798519	6	50,788,778	C	A	0.0251	0.0023	4.78189 × 10^−27^	0.211526	−9.83 × 10^−05^	0.000176144	0.580000008596693	NA	119.094517958412
rs3803487	15	61,189,609	A	G	0.0235	0.002	1.01391 × 10^−30^	0.320949	−2.05 × 10^−06^	0.000149366	0.989999989519613	NA	138.0625
rs3842763	11	2,179,204	T	G	−0.017	0.0021	2.073 × 10^−15^	0.30375	−4.11 × 10^−05^	0.000160032	0.800000023961726	NA	65.5328798185941
rs399322	3	64,793,523	C	T	0.0265	0.0021	7.09578 × 10^−36^	0.62336	8.47437 × 10^−05^	0.000135273	0.530000159135322	NA	159.240362811791
rs4053127	6	9,531,791	CAT	C	−0.0134	0.0021	3.16097 × 10^−10^	0.679931	−6.98 × 10^−05^ × 10^−83^	0.000139156	0.620000443273307	NA	40.7165532879819
rs4253536	10	81,374,457	C	T	0.0265	0.0044	2.053 × 10^−09^	0.073407	−0.000339411	0.000260869	0.190000174579693	NA	36.2732438016529
rs428463	2	159,964,516	C	T	−0.0114	0.002	2.48102 × 10^−08^	0.685397	9.81387 × 10^−05^	0.000144473	0.499999995007974	NA	32.49
rs4338470	10	76,233,448	A	G	−0.0175	0.0021	1.97606 × 10^−17^	0.635841	4.94377 × 10^−05^	0.000153148	0.749999545138786	NA	69.4444444444445
rs4359266	12	42,743,598	T	C	−0.0143	0.0019	1.14393 × 10^−13^	0.588756	−2.57 × 10^−05^	0.000137404	0.849999949672056	NA	56.6454293628809
rs4410790	7	17,284,577	C	T	−0.0141	0.002	7.36207 × 10^−13^	0.556968	2.10251 × 10^−05^	0.000140099	0.880000056431416	NA	49.7025
rs4461138	17	44,987,045	G	T	−0.0272	0.002	2.13206 × 10^−42^	0.572093	−0.000199418	0.000143233	0.160000006389794	NA	184.96
rs4471322	1	217,531,786	T	G	0.0144	0.0023	7.20991 × 10^−10^	0.788459	0.000152423	0.000160692	0.340000064945877	NA	39.1984877126654
rs4483062	7	29,570,901	T	C	−0.013	0.0024	3.50002 × 10^−08^	0.206743	5.66745 × 10^−05^	0.000166019	0.730000235121762	NA	29.3402777777778
rs4567978	2	54,570,557	G	A	−0.0132	0.0023	1.13799 × 10^−08^	0.787779	4.04592 × 10^−05^	0.000170879	0.809999964789546	NA	32.937618147448
rs4605486	3	66,818,706	G	A	0.0108	0.0019	2.223 × 10^−08^	0.552898	−0.000128464	0.000137922	0.349999964257903	NA	32.3102493074792
rs4731532	7	128,572,766	A	G	0.0114	0.002	2.28202 × 10^−08^	0.399556	−0.000296564	0.000135311	0.0280001269243453	NA	32.49
rs4739760	8	81,811,312	C	T	0.0132	0.0019	7.0583 × 10^−12^	0.445995	6.11881 × 10^−05^	0.000138229	0.660000097957427	NA	48.2659279778393
rs4766566	12	111,706,877	T	C	−0.0129	0.0021	1.61102 × 10^−09^	0.378151	−9.14 × 10^−05^	0.000156085	0.559999965176919	NA	37.734693877551
rs4845945	1	10,734,335	A	G	0.0259	0.0039	1.78813 × 10^−11^	0.0663255	0.000619656	0.000346307	0.0739997069733888	NA	44.10322156
rs4906214	14	102,995,668	A	C	−0.0144	0.0023	5.35205 × 10^−10^	0.55076	−0.000153574	0.00013626	0.259999791679655	NA	39.1984877126654
rs4957107	5	699,647	A	G	−0.0206	0.0031	4.35813 × 10^−11^	0.18691	−6.43 × 10^−05^	0.000184853	0.730000235121762	NA	44.1581685744017
rs4966019	15	99,274,326	T	C	−0.0225	0.002	1.27791 × 10^−30^	0.595023	−0.000221057	0.000141104	0.119999932014548	NA	126.5625
rs496908	2	219,357,742	T	A	0.0191	0.0023	2.76694 × 10^−16^	0.56729	0.000189059	0.000137215	0.170000030775649	NA	68.9621928166351
rs529224	1	100,818,178	C	G	0.011	0.0019	1.704 × 10^−08^	0.401873	6.9145 × 10^−05^	0.000139934	0.620000443273307	NA	33.5180055401662
rs55982832	7	55,050,103	A	G	0.0156	0.0028	2.516 × 10^−08^	0.21345	7.12263 × 10^−05^	0.00016482	0.67000030437982	NA	31.0408163265306
rs56019566	2	15,788,692	T	C	−0.0154	0.002	5.3064 × 10^−15^	0.423634	−1.88 × 10^−05^	0.000141745	0.889999986382575	NA	59.29
rs56059584	10	43,889,205	G	A	0.0156	0.0024	1.47999 × 10^−10^	0.185297	0.000104771	0.000174281	0.550000393231399	NA	42.25
rs56205943	12	57,679,414	A	G	−0.0296	0.0024	1.59698 × 10^−33^	0.193628	−9.03 × 10^−05^	0.000158438	0.570000189426062	NA	152.111111111111
rs57711468	22	41,774,256	C	T	−0.0145	0.0023	2.45601 × 10^−10^	0.48426	−0.000181454	0.000135253	0.180000205117632	NA	39.7448015122873
rs58483230	16	69,519,398	C	T	−0.021	0.0038	2.21498 × 10^−08^	0.0700215	−9.24 × 10^−05^	0.000255882	0.719999176986299	NA	30.5401662049862
rs58856116	16	51,688,580	G	A	0.0124	0.0021	5.815 × 10^−09^	0.273395	0.000257482	0.000152633	0.092000460251551	NA	34.8662131519274
rs5951348	23	100,784,211	A	C	0.0123	0.0017	2.40713 × 10^−13^	0.392619	−0.00017065	0.000124141	0.170000030775649	NA	52.3494809688582
rs6025101	20	55,289,160	A	G	0.0121	0.002	6.63193 × 10^−10^	0.592993	−5.08 × 10^−05^	0.000140937	0.719999176986299	NA	36.6025
rs6055748	20	8,315,317	G	A	−0.0147	0.0025	3.13502 × 10^−09^	0.217705	3.15277 × 10^−06^	0.000148061	0.980000009720029	NA	34.5744
rs60910743	17	80,189,813	A	G	0.0228	0.0024	2.12618 × 10^−21^	0.218734	0.000192923	0.0001592	0.230000086844209	NA	90.2500000000001
rs61963240	13	111,096,037	G	T	0.0193	0.0025	2.01002 × 10^−14^	0.198551	0.000135759	0.000151423	0.370000235082988	NA	59.5984
rs620088	11	65,508,986	A	G	0.0162	0.002	1.16198 × 10^−15^	0.32948	−0.000155978	0.000141169	0.27000014662192	NA	65.61
rs62083214	18	3,640,786	T	C	−0.0233	0.0034	5.0038 × 10^−12^	0.13517	−8.20 × 10^−05^	0.000199056	0.680000136680911	NA	46.9628027681661
rs62275515	3	168,725,418	G	A	0.0152	0.002	3.93913 × 10^−14^	0.400354	0.000211058	0.00013523	0.119999932014548	NA	57.76
rs62400344	6	24,180,690	C	T	0.0129	0.0021	6.58901 × 10^−10^	0.295457	−2.17 × 10^−05^	0.000150476	0.889999986382575	NA	37.734693877551
rs62411644	4	18,172,771	G	A	−0.0279	0.0047	4.343 × 10^−09^	0.062826	−0.000491484	0.000281182	0.0800000023961726	NA	35.2381167949298
rs62466318	7	73,042,085	T	C	−0.0176	0.0025	3.30293 × 10^−12^	0.17518	−0.000140472	0.000168771	0.410000135265173	NA	49.5616
rs62473663	7	77,216,415	T	C	0.016	0.0022	1.92619 × 10^−13^	0.263762	2.79505 × 10^−05^	0.000148223	0.849999949672056	NA	52.8925619834711
rs62505275	8	30,437,639	T	C	0.018	0.0023	1.6151 × 10^−14^	0.221655	−0.000194429	0.000152592	0.20000000199681	NA	61.2476370510397
rs67387640	2	160,675,638	T	C	−0.0128	0.002	2.80698 × 10^−10^	0.342463	−0.000291327	0.000141431	0.0389995862874433	NA	40.96
rs6757766	2	121,998,898	T	C	−0.0354	0.0033	1.87802 × 10^−26^	0.13877	−0.000232741	0.000195742	0.230000086844209	NA	115.074380165289
rs6770483	3	79,594,835	C	T	−0.0112	0.002	2.82599 × 10^−08^	0.668148	1.94188 × 10^−05^	0.000144915	0.889999986382575	NA	31.36
rs6793835	3	135,819,934	A	G	−0.0282	0.0026	3.74628 × 10^−27^	0.26437	−0.000117884	0.000154041	0.440000327764078	NA	117.639053254438
rs6969701	7	106,628,948	C	T	−0.0147	0.0022	5.43 × 10^−11^	0.750739	−4.62 × 10^−05^	0.000166391	0.780000713574729	NA	44.646694214876
rs6978677	7	75,636,240	C	T	0.0114	0.0021	2.82098 × 10^−08^	0.667818	2.06591 × 10^−05^	0.000152993	0.889999986382575	NA	29.469387755102
rs6998726	8	127,495,576	A	G	−0.0261	0.0021	1.33598 × 10^−35^	0.650492	2.78276 × 10^−05^	0.000153757	0.860000096548623	NA	154.469387755102
rs700750	7	46,753,491	A	C	0.0154	0.0021	3.51722 × 10^−13^	0.694451	4.07259 × 10^−05^	0.000139943	0.770000487266739	NA	53.7777777777778
rs703031	10	29,203,987	T	C	−0.0275	0.0028	7.44903 × 10^−23^	0.146137	−0.000101228	0.000174033	0.559999965176919	NA	96.4604591836735
rs7096540	10	13,495,241	C	T	−0.0113	0.002	3.20701 × 10^−08^	0.407761	3.69636 × 10^−05^	0.000147358	0.800000023961726	NA	31.9225
rs7202316	16	719,258	C	T	0.0136	0.0024	1.39399 × 10^−08^	0.544311	−0.000123081	0.000141979	0.39000035289358	NA	32.1111111111111
rs7298123	12	56,860,073	T	C	−0.0264	0.0025	1.38899 × 10^−25^	0.827219	−0.000126471	0.000169032	0.450000503808422	NA	111.5136
rs7365304	1	16,371,510	A	G	0.0158	0.0021	6.1759 × 10^−14^	0.290201	0.000200232	0.000146634	0.170000030775649	NA	56.6077097505669
rs75342066	8	66,644,963	G	T	0.0206	0.0037	2.97598 × 10^−08^	0.0711009	5.19911 × 10^−05^	0.000244729	0.830000014570433	NA	30.9978086194302
rs7564041	2	166,734,933	G	T	0.0105	0.0019	4.21105 × 10^−08^	0.483022	4.34792 × 10^−05^	0.000136897	0.749999545138786	NA	30.5401662049862
rs7576384	2	113,993,385	G	C	0.0307	0.002	2.1301 × 10^−53^	0.356014	−1.17 × 10^−05^	0.000141862	0.929999896026408	NA	235.6225
rs76273615	3	169,092,038	G	A	0.0391	0.0027	3.57108 × 10^−47^	0.145058	0.000150505	0.000207118	0.470000153744122	NA	209.713305898491
rs76369738	4	23,768,836	T	C	−0.0309	0.0052	2.74897 × 10^−09^	0.0380046	−0.000389176	0.000368462	0.290000001402971	NA	35.3110207100592
rs7702068	5	67,748,263	A	G	0.0201	0.0035	1.41599 × 10^−08^	0.0810018	−5.71 × 10^−05^	0.000219261	0.78999983393885	NA	32.9804081632653
rs7735249	5	53,310,139	G	C	−0.0252	0.0036	5.17249 × 10^−12^	0.11273	−1.15 × 10^−05^	0.000215514	0.959999926966642	NA	49
rs7766720	6	107,172,979	C	T	0.0216	0.0031	1.80011 × 10^−12^	0.109067	−0.000172113	0.000249933	0.489999909706502	NA	48.5494276795005
rs77777887	12	47,180,881	T	A	0.0292	0.0039	6.30957 × 10^−14^	0.0634713	0.000525846	0.000268031	0.0499999995007974	NA	56.0578566732413
rs77924615	16	20,392,332	A	G	−0.0371	0.0024	4.75554 × 10^−55^	0.203629	0.000209014	0.000171622	0.220000161685546	NA	238.960069444445
rs7834797	8	23,759,535	A	G	0.0272	0.002	1.01789 × 10^−40^	0.463512	0.000273678	0.000136957	0.0460002296665068	NA	184.96
rs7873103	9	109,644,110	A	C	0.0117	0.002	1.128 × 10^−08^	0.317923	0.000225833	0.000146481	0.119999932014548	NA	34.2225
rs79449986	17	46,559,195	T	A	−0.0363	0.0062	4.05303 × 10^−09^	0.038581	−0.000404949	0.000366981	0.27000014662192	NA	34.2791363163371
rs79958663	10	95,910,761	T	C	−0.0148	0.0025	3.63396 × 10^−09^	0.177707	0.00019414	0.000181735	0.290000001402971	NA	35.0464
rs80282103	10	899,071	T	A	0.0296	0.0035	1.60916 × 10^−17^	0.0825627	−1.82 × 10^−05^	0.000245944	0.940000100430209	NA	71.5232653061225
rs8098626	18	45,667,057	G	C	0.0198	0.002	3.50913 × 10^−23^	0.650478	0.000105026	0.000141531	0.460000178281084	NA	98.01
rs836968	12	50,267,335	T	C	0.0175	0.0021	2.47799 × 10^−17^	0.353548	0.000218155	0.000153277	0.149999910525364	NA	69.4444444444445
rs838133	19	49,259,529	G	A	0.0409	0.0024	1.95884 × 10^−66^	0.54815	−2.26 × 10^−06^	0.000139901	0.989999989519613	NA	290.418402777778
rs889400	16	69,555,876	T	A	−0.0189	0.002	3.55877 × 10^−21^	0.641658	1.39228 × 10^−06^	0.000144859	0.989999989519613	NA	89.3025
rs9306894	2	20,878,105	G	A	0.0138	0.002	1.61696 × 10^−12^	0.420067	−3.38 × 10^−05^	0.000140893	0.809999964789546	NA	47.61
rs9316500	13	51,094,114	G	T	0.0167	0.002	1.33999 × 10^−16^	0.380376	−1.05 × 10^−05^	0.000148022	0.940000100430209	NA	69.7225
rs9482771	6	127,446,610	C	G	0.0314	0.0019	2.53688 × 10^−61^	0.501819	0.00023683	0.00013535	0.0800000023961726	NA	273.119113573407
rs9517448	13	99,438,618	C	A	0.019	0.0025	1.51112 × 10^−14^	0.195598	0.000119836	0.00020255	0.550000393231399	NA	57.76
rs9529913	13	72,345,089	T	C	0.0263	0.0021	8.32914 × 10^−36^	0.464855	−4.48 × 10^−05^	0.000138341	0.749999545138786	NA	156.845804988662
rs9594689	13	42,737,268	G	T	−0.0111	0.0019	1.40301 × 10^−08^	0.425222	0.000180096	0.000140713	0.20000000199681	NA	34.1301939058172
rs963837	11	30,749,090	C	T	−0.0365	0.0019	2.79576 × 10^−79^	0.4228	−9.60 × 10^−05^	0.000135745	0.479999737642877	NA	369.04432132964
rs9880162	3	187,719,348	G	A	−0.054	0.002	2.09894 × 10^−153^	0.311432	−0.000147825	0.000146253	0.310000218541595	NA	729

Site ID; *F*: *F*-statistic (measures the strength of instrumental variables (IV)), *F* >10 shows that the instrumental variables are effective; *P*-value: If the value is less than 5 × 10⁻⁸, it indicates a significant genome-wide association.

EA/OA = effect allele/other allele, EAF = allele frequency, NA = not applicable, SE = standard error, SNP = single nucleotide polymorphism.

### 2.3. MR analysis

In this MR analysis, inverse variance weighted (IVW), weighted median (WME) and MR Egger regression were used to assess the causal relationship between BUN and BC. The IVW method is an important approach in MR analysis, which is unique in that it does not require consideration of intercept terms and uses the reciprocal of the outcome variance as a weight for fitting, thus eliminating the need to consider horizontal pleiotropy. However, the accuracy of the IVW method is affected by the quality of the included SNPs. When the SNPs included in the MR analysis strictly meet the screening criteria, fixed effects IVW can provide precise causal relationships. WME weights the causal relationship results of different genetic variations and takes the median. Its characteristic is that the validity requirements for SNPs are relatively relaxed, and even with only 50% of valid SNPs, WME can still obtain meaningful results. The MR Egger regression method also uses the reciprocal of the outcome variance as a weight fitting, but it takes into account the existence of the intercept term in the analysis. Therefore, when the intercept term value is 0, the results of IVW and MR Egger regression methods are equivalent. Therefore, this study takes the analysis results of IVW as the main reference, and the analysis results of WME and MR Egger regression as reference and supplement. At the same time, this study will also conduct reverse MR analysis, with SNPs closely related to BC as exposure factors and BUN level as outcome variables, to further explore the reverse causal relationship between BC and BUN.

### 2.4. Sensitivity analysis

Sensitivity analysis is the process of conducting horizontal pleiotropy and heterogeneity tests on the causal relationships of MR analysis to ensure the reliability of the analysis results. To avoid differences between data, Cochran Q statistics were used for heterogeneity testing. When *P* >.05, it indicates the absence of heterogeneity; The MR Egger intercept term is one of the methods used for horizontal testing. When *P* >.05, it indicates the absence of horizontal pleiotropy; otherwise, there is horizontal pleiotropy; MR-PRESSO test is also a method for testing horizontal pleiotropy. Unlike the MR Egger intercept term, MR-PRESSO test can also screen for outliers. When outliers exist, they need to be removed and IVW analysis needs to be performed again to ensure the robustness of the results; The “leave one method” results are presented in a forest plot, and its principle is to remove SNPs one by one and use the remaining SNPs for causal evaluation. When there is a significant bias in the results after removing a SNP, the sensitivity is higher.

### 2.5. Statistical methods

This study used the “Two Sample MR” and “MR-PRESSO” R packages in R software version 4.4.1 for online import of IV, F-value calculation, instrumental variable screening, MR analysis, and sensitivity analysis. BC was set as the outcome variable, and the effect value was a binary variable, which was expressed by OR and 95% CI. *P* <.05 is considered statistically significant.

## 3. Results

### 3.1. Selection of instrumental variables

This study removed 1 incompatible SNP (rs12476098) and removed 27 palindromic sequences (rs10766469, rs1113499, rs12151866, rs12247543, rs12476098, rs13032786, rs13230509, rs162004, rs17398736, rs17663555, rs187355703, rs2055685, rs2596398, rs3212531, rs347593, rs35601737, rs35610898, rs496908, rs529224, rs7576384, rs7735249, rs7777887, rs79449986, rs80282103, rs8098626, rs889400, rs9482771), 178 SNPs were ultimately included, and after testing, the F-values of all included SNPs were >10, indicating that there was no bias in the results caused by weak IV.

### 3.2. MR analysis

**Figure 2.**
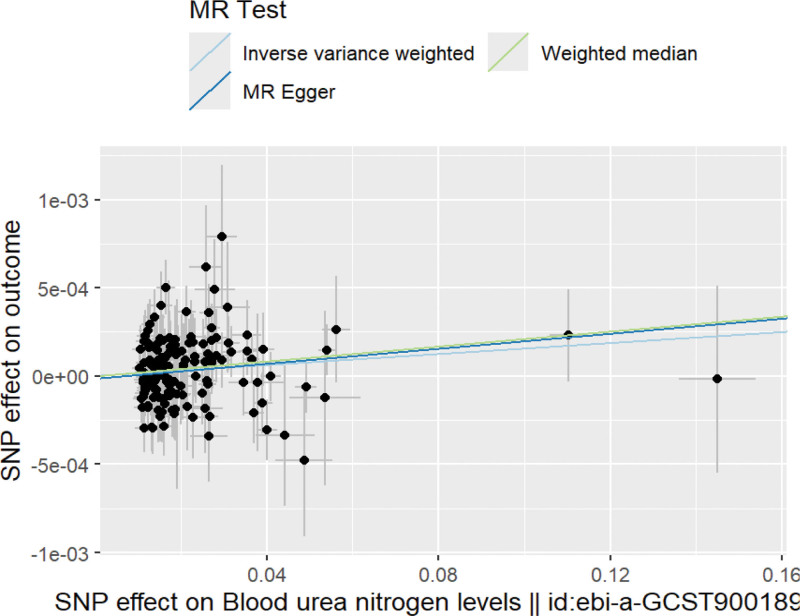
Scatter chart of MR analysis results of BUN and BC. Note: X-axis: SNP effects on BUN; Y-axis: SNP effects on outcome variables.As shown in the figure, there is a positive correlation between the predicted blood urea nitrogen level and the risk of BC. BC = bladder cancer, BUN = blood urea nitrogen, MR = Mendelian randomization, SNP = single nucleotide polymorphism.

IVW analysis showed that there was a causal relationship between BUN and BC (OR = 1.0016, 95% CI: 1.0004~1.0027, *P* < .05); WME analysis results verified the IVW results, and also confirmed that the increased BUN level would increase the risk of BC (OR = 1.0021, 95% CI:, 1.0002~1.0040, *P* < .05); MR Egger regression method did not confirm that there is a causal relationship between exposure and outcome (OR = 1.0021, 95% CI: 1.0000–1.0047, *P* = .11), but the scatter plot results (Fig. 2) show that the selected working variables are stable, and the slopes of the 3 analysis methods are positive, so it can still be considered that there is a causal relationship between them. We conducted a reverse MR analysis, treating BC-related SNPs as IV and BC as the exposure factor with BUN as the outcome variable. However, empirical evidence showed no reverse causality between the 2 (*P* >.05). In conclusion, there exists a positive causal relationship between BUN levels and BC. Elevated BUN levels can increase the risk of developing BC.

### 3.3. Sensitivity analysis

**Figure 3.**
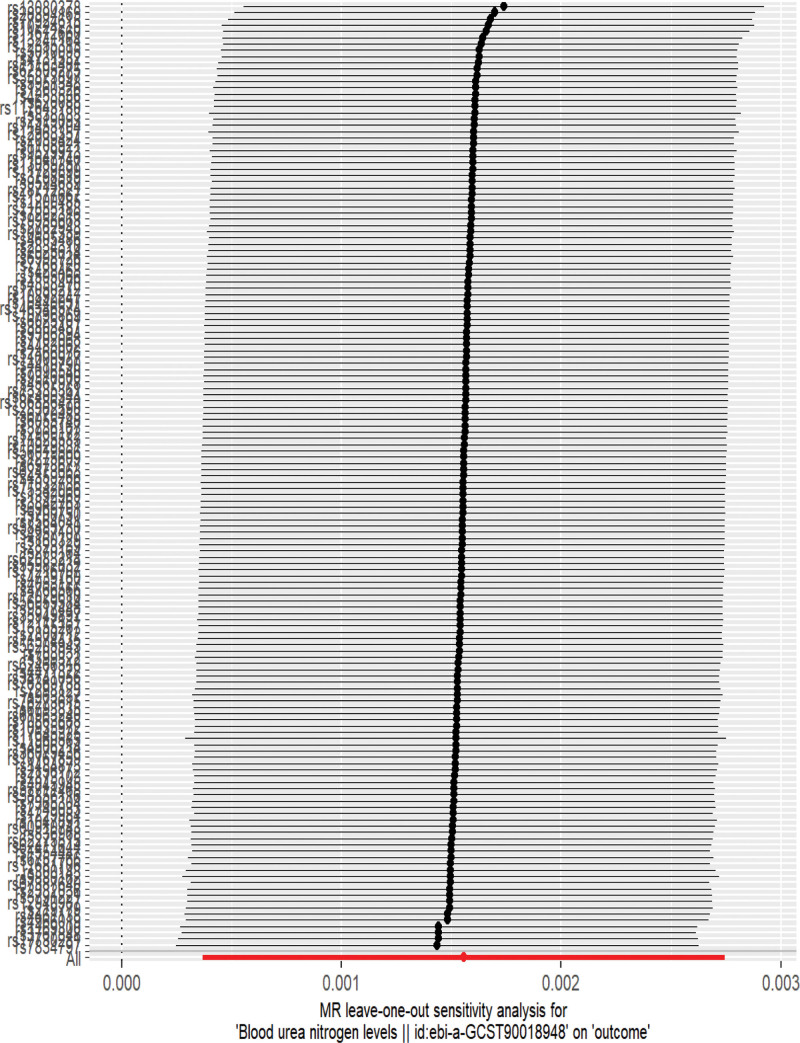
Analysis of BUN and BC. Note: The leave-one-out sensitivity analysis confirmed the robustness of the results. BC = bladder cancer, BUN = blood urea nitrogen, MR = Mendelian randomization.

**Figure 4.**
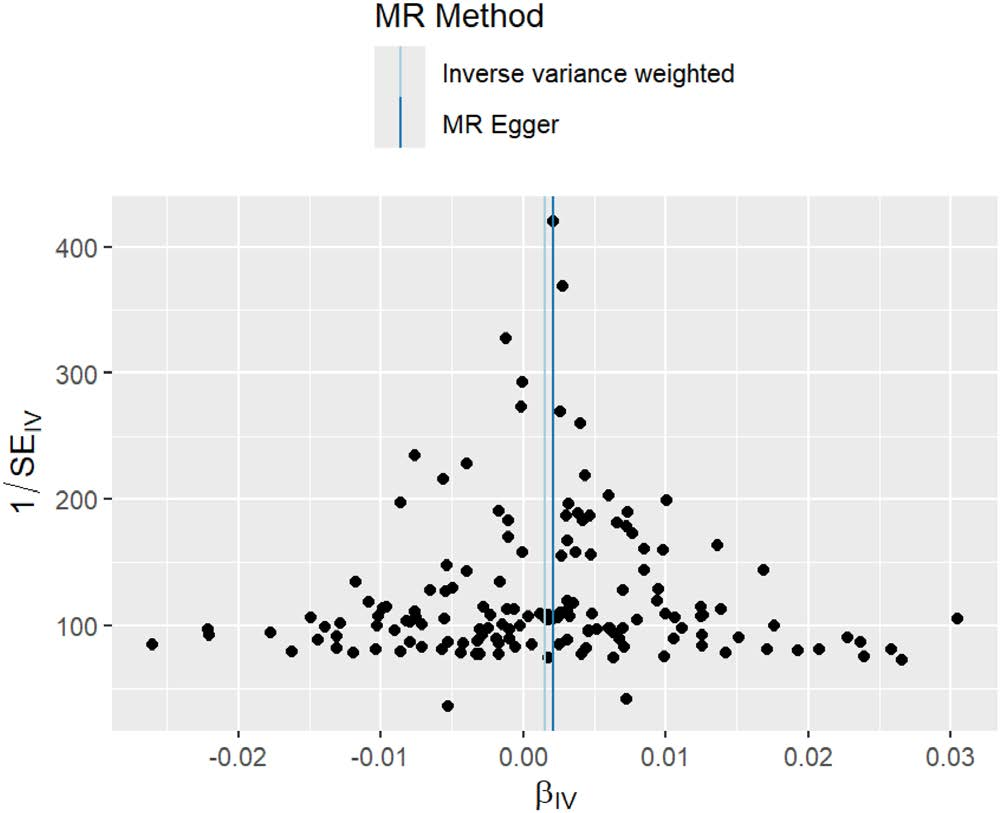
Funnel diagram of BUN and BC MR analysis. Note: This funnel plot illustrates the relationship between the OR of each SNP’s effect on BC and its precision (1/SE). The scattered points are symmetrically distributed on both sides of the fitted lines, suggesting limited likelihood of horizontal pleiotropy influencing the primary analysis results. BC = bladder cancer, BUN = blood urea nitrogen, MR = Mendelian randomization, OR = odds ratio, SE = standard error, SNPs = single nucleotide polymorphisms.

Using the PRESSO test and MR Egger intercept term to test for level pleiotropy, the PRESSO test results showed *P* = .36 and no outliers were detected; The MR Egger intercept results showed *P* = .63, therefore there is no horizontal pleiotropy in this study. The heterogeneity test results showed that Cochran Q = 155.2202, *P* = .37, indicating the absence of heterogeneity. The “leave one method” (Fig. 3) showed that there was no SNP bias in the results after removing SNPs one by one. The vertical line in the funnel plot (Fig. 4) represents the IVW line, and the points represented by SNPs on both sides of the IVW line are symmetrically distributed, indicating stable results and a low possibility of bias.

## 4. Discussions

The urea cycle is the process in which ammonia is converted into urea in animals, mainly mediated by urea cycle enzymes stored in liver mitochondria and cytoplasm. The generated urea is filtered by the glomerulus, reabsorbed by the renal tubules, and excreted through urine. The urea content in arterial blood is called BUN, and its level depends on protein intake from food, tissue protein breakdown metabolism, and liver and kidney function. In clinical practice, BUN is often used as an indicator to evaluate renal function. In recent years, scholars have continuously studied and found that BUN has predictive value for cardiovascular diseases,^[6,7]^ respiratory diseases,^[8]^ and neurological diseases.^[9]^ This study analyzed the causal relationship between BUN level and BC based on MR method, and found that the 2 were positively correlated. When BUN level increased, the risk of BC also increased.

BC is one of the top ten cancers with high incidence rate in the world, and is also the most common tumor of the urinary system. It has a high incidence rate, is difficult to cure, and is easy to relapse. The early symptoms of the disease are hidden, some patients may show painless hematuria, and some patients may have no obvious clinical manifestations.^[10]^ The examination of BC includes urine cytological analysis, detection of urine tumor markers, cystoscopy and imaging examination. Cystoscopy is one of the important methods used to diagnose BC.^[11]^ This examination is invasive and not a routine item of annual physical examination. Most people go to the hospital only when they have obvious symptoms, often missing the best treatment period, causing irreversible damage, even distant metastasis, and life-threatening. Data shows that in 2018, 549,393 patients worldwide were diagnosed with BC, and 199,922 patients died of BC.^[12]^ At present, scholars at home and abroad focus on the study of risk factors and treatment techniques of BC. Previous studies have shown that the main risk factor for BC is smoking, which may be excreted from the body through urine metabolism with tobacco metabolites.^[13]^ In this process, the urothelium has long-term contact with metabolic carcinogens, further causing canceration. In addition, the elderly, male, obesity, long-term urine holding, hair dye use, and environmental toxin exposure are also considered as risk factors for BC,^[14–16]^ but most of them are observational or randomized controlled trials. A high-quality research often needs to invest a lot of time, manpower and material resources. In addition, because BC patients have more late complications and complicated conditions, the research process cannot exclude the interference of mixed factors, which inevitably leads to biased research results. MR is a recently emerged experimental method based on natural randomization of the human genome, widely used to evaluate the causal relationship between exposure and disease. Compared with traditional experimental methods, MR greatly avoids the influence of confounding factors and result bias caused by reverse causality.

In this study, we selected 3 MR analysis methods, of which IVW and WME showed that there was a causal relationship between BUN level and BC, and the MR Egger method did not get the result that there was a causal relationship between the 2, but we still draw the above conclusions based on the following reasons: the accuracy of the IVW method mainly depends on the strong correlation between the SNPs included and the exposure factors, that is, BUN. When selecting the IV, we strictly abide by the 3 assumptions of MR, set the standard threshold *P* < 5 × 10-8, removed the linkage imbalance, and finally selected the IV with F-values >10, ensuring that the selected SNPs have genetic independence and BUN N has strong correlation, which lays the foundation for the robustness of IVW results. The MR Egger regression method is mainly used to test and correct horizontal pleiotropy. When there is horizontal pleiotropy in the results, the MR Egger regression method is preferred. However, in this study, when using the MR Egger intercept term and PRESSO test for horizontal pleiotropy, P values >0.05 indicate that there is no horizontal pleiotropy in the analysis results. Therefore, the MR Egger regression method is only used as a reference. The scatter plot shows that the slopes of the 3 analysis methods are positive, indicating that there is a positive relationship between BUN and BC. To sum up, the results of this study: there is a causal relationship between BUN level and BC, and the conclusion that increased BUN level will increase the risk of BC is valid.

## 5. Strengths

Compared with traditional research methods, this study utilized MR to analyze the relationship between exposure factors and outcome variables from a genetic perspective, avoiding the bias caused by confounding factors and reverse causality on the results. In addition, the GWAS data selected for this study are all from publicly available databases and do not require additional ethical review, saving time and manpower. And after searching the Chinese and English databases, no scholar has found that this method has been used to study the relationship between BUN level and BC. This study supplements this gap. The results of this study show that strict control of BUN level can reduce the risk of BC.

## 6. Limitations

This study also has relative limitations. Because the included races are all European, although it avoids the result deviation caused by population, it also limits the applicability of this study result. The causal relationship between BUN level of other ethnic groups and BC is unknown. MR research emphasizes the role of genes while ignoring the influence of environmental factors, such as dietary habits, lifestyle, and work environment, which can all have an impact on outcomes. In addition, the exposure factors included in this study, namely the IV strongly associated with BUN levels, were not stratified by gender, which may have an impact on the analysis results.

## 7. Conclusion

This study demonstrates a causal relationship between elevated BUN levels and increased BC risk in European populations through a dual-sample MR analysis. The findings not only provide new metabolic evidence for understanding BC etiology but also identify potential biomarkers for high-risk population screening and personalized treatment. While limitations exist regarding ethnicity and environmental factors, this research lays the groundwork for future interdisciplinary and multi-dimensional investigations. Subsequent high-quality studies are needed to further validate these conclusions and advance clinical translation.

## Acknowledgments

All authors express their profound gratitude to scholars Sakaue S and Burronss for their data support, and to all collaborators and participants who took part in this study.

## Author contributions

**Data curation**: Jinming Bai.

**Writing – original draft**: Jinming Bai.

**Writing – review & editing**: Lingling Wang.

**Supervision**: Lingling Wang.

**Funding acquisition**: Lingling Wang.
